# *In silico* analysis of alternative splicing events implicated in intracellular trafficking during B-lymphocyte differentiation

**DOI:** 10.3389/fimmu.2022.1030409

**Published:** 2022-11-10

**Authors:** Felix Ostwaldt, Bruna Los, Florian Heyd

**Affiliations:** Laboratory of RNA Biochemistry, Institute of Chemistry and Biochemistry, Freie Universität Berlin, Berlin, Germany

**Keywords:** B cell differentiation, memory B cell, antibody-secreting cells, secretory pathway, alternative splicing, COPII, NERF/ELF2

## Abstract

There are multiple regulatory layers that control intracellular trafficking and protein secretion, ranging from transcriptional to posttranslational mechanisms. Finely regulated trafficking and secretion is especially important for lymphocytes during activation and differentiation, as the quantity of secretory cargo increases once the activated cells start to produce and secrete large amounts of cytokines, cytotoxins, or antibodies. However, how the secretory machinery dynamically adapts its efficiency and specificity in general and specifically in lymphocytes remains incompletely understood. Here we present a systematic bioinformatics analysis to address RNA-based mechanisms that control intracellular trafficking and protein secretion during B-lymphocyte activation, and differentiation, with a focus on alternative splicing. Our *in silico* analyses suggest that alternative splicing has a substantial impact on the dynamic adaptation of intracellular traffic and protein secretion in different B cell subtypes, pointing to another regulatory layer to the control of lymphocyte function during activation and differentiation. Furthermore, we suggest that NERF/ELF2 controls the expression of some COPII-related genes in a cell type-specific manner. In addition, T cells and B cells appear to use different adaptive strategies to adjust their secretory machineries during the generation of effector and memory cells, with antibody secreting B cell specifically increasing the expression of components of the early secretory pathway. Together, our data provide hypotheses how cell type-specific regulation of the trafficking machinery during immune cell activation and differentiation is controlled that can now be tested in wet lab experiments.

## Introduction

During the course of a T cell-dependent B cell activation, naïve B cells can proliferate and differentiate into antibody-secreting cells (ASC) or memory B cells (MBC). MBCs and ASC are responsible for the defense against and the long-term humoral immunity generated by infections and many vaccines (1). MBCs are long-lived cells and are responsible for a quick reaction upon antigen re-challenge. These cells express cell-surface antibodies but do not secrete them (1). MBCs can differentiate into ASC after exposure to the same exogenous stimuli (2). ASCs include short-lived plasmablasts (PB) and long-lived plasma cells (PC) and are responsible for antibody-mediated immunity (2). During differentiation and maturation, ASCs expand their cytoplasm, endoplasmic reticulum (ER), and Golgi apparatus, allowing an increased synthesis and secretion of antibodies (3, 4). From the ER, immunoglobulin (Ig) molecules are packaged first into COPII-coated vesicles and transported to the Golgi apparatus. In the Golgi, Igs undergo glycosylation and are then further transported towards the plasma membrane (5).

In eukaryotic life, intracellular trafficking is essential for generating and maintaining organelles, and for the communication of cells with their surroundings. The transport from the ER through the Golgi to the plasma membrane and subsequent exocytosis is a highly regulated process. The basis of these transport mechanisms throughout evolution is vesicular transport, which also enables sorting processes during endo- and exocytosis to generate cargo specificity (6, 7). The big picture of intracellular transport becomes more and more detailed through high-resolution imaging techniques, which, for example, suggested that tubular structures rather than classical vesicles mediate or contribute to ER to the Golgi transport (8, 9).

The classical model of the COPII coat consists of an outer and inner layer. The outer layer consists of *SEC13* and *SEC31*, which form the coat, and give it its rigidity and structure. SEC23 and SEC24 create the inner layer, where SEC24 with four isoforms (A-D) is the major cargo binder and sorter (10–12), and SEC23 together with SEC31 regulates the GTPase activity of SAR1, which is responsible for curving the ER membrane (13). Additional regulators are SEC16, which functions as a scaffold protein that recruits all other members of the COPII coat to ER exit sites (ERES) and is actually used to define ERES (14). Kelch-like protein 12 (*KLHL12*) is another important member of the COPII machinery and is especially required for the transport of larger cargoes. Together with CUL, it is responsible for ubiquitinating SEC31 (A or B) (15). This allows the outer layer of the COPII coat to be more flexible and enables large cargoes like collagen to exit the ER (16).

Clathrin-mediated endocytosis is the classical way in which the cell brings specific cargoes into the cytoplasm. Clathrin heavy chains trimerizise into triskelia. These are the de facto monomers, out of which clathrin cages will form (17). For that to happen, triskelia have to come into close proximity through adaptor proteins like *AP2* or *PICALM*. These adaptors bind at least two triskelia and bring them together. Two binding sites on each adaptor bind each a separate triskelia, supporting their polymerization into a clathrin cage (18). The distance between these binding sites seems to be crucial. A shorter distance of the linker between the binding sites promotes inter-triskelia binding, longer linker promotes intra-skelia binding, which can regulate clathrin-dependent endocytosis (19).

Post-transcriptional regulation of gene expression is essential for modulating the transcriptome and proteome of ASCs (3). One important mechanism to increase proteome diversity and to control cellular functionality is alternative splicing (AS). In the past years, many studies have identified changes in AS during lymphocyte activation that are important for the proper functioning of the adaptive immune system (20–23). As most of these studies have focussed on T cell activation, the impact of AS on B cell activation is less well understood, and even less is known about the impact of AS during different stages of B cell differentiation and maturation. Furthermore, while increased secretory capacity can be expected from antibody-secreting B cell subtypes, a systematic analysis of the secretory machinery during B cell differentiation is missing. Here we address transcriptional and post-transcriptional changes that control protein secretion during B cell differentiation and provide evidence for global adaption of the secretory machinery through changes in gene expression and alternative splicing. Our study is entirely based on a bioinformatics approach as a first step to generate and discuss hypotheses, which will allow wet lab validations in future research.

## Material and methods

### Differential gene expression analysis

We analyzed publicly available RNA-seq data for B cell differentiation (GSE148924) and human tissues (PRJEB4337). Reads were aligned to the human genome (GRCh38/hg38) using Salmon (v1.8.0) on default settings. Gene counting was then performed with featureCounts (version 1.6.4) and the resulting count matrix was used as input for DESeq2 (version 1.28.1) (24). Differential gene expression analysis was performed by running DESeq2 with fitType = “local”. Log2 fold change (FC) shrinkage was performed by the adaptive shrinkage algorithm to reduce the effect of low expression levels on fold changes. Significantly differential genes were identified by filtering on Benjamini-Hochberg adjusted *P*-value (*P*adj) < 0.001 and absolute log2FC > 0.5 (or < -0.5). For heatmaps, mean normalized expression counts were calculated for each condition. Pairwise correlation of columns was performed using Pearson. Data analysis was performed using Python 3.7.11 with scientific libraries pandas (25), NumPy (26), matplotlib (27), and seaborn. Volcano plots and dot plots were generated using R v. 4.2.1. with libraries tidyverse, ggrepel, dplyr, and ggplot2.

### Differential alternative splicing analysis

For alternative splicing (AS) analysis reads were aligned to the human genome (GRCh38/hg38) using STAR (version 2.7.5b) on default settings. Differential AS events were determined using rMATS 4.1.2 (28). Sashimi blots were created using the IGV genome browser.

### Gene ontology enrichment analysis

Gene ontology (GO) term enrichment was performed on gene symbols using the enrichGO function in R v. 4.2.1. with libraries clusterProfiler (29, 30), org.Hs.eg.db (31), and AnnotationDbi (32). Only GO enrichments with a false discovery rate (FDR) < 0.05 are presented.

### Enriched transcription factor binding motif analysis

The ShinyGO 0.76 tool (http://bioinformatics.sdstate.edu/go/) was used to identify transcription factor (TF) binding motifs enriched in the promoters of target genes.

### Protein structure prediction

The protein 3D structure of the different isoforms was predicted using Robetta (https://robetta.bakerlab.org/) and visualized using PyMol v.2.3.4.

## Results

### Differential expression of trafficking-related genes in memory-B-cells

**Figure 1:**
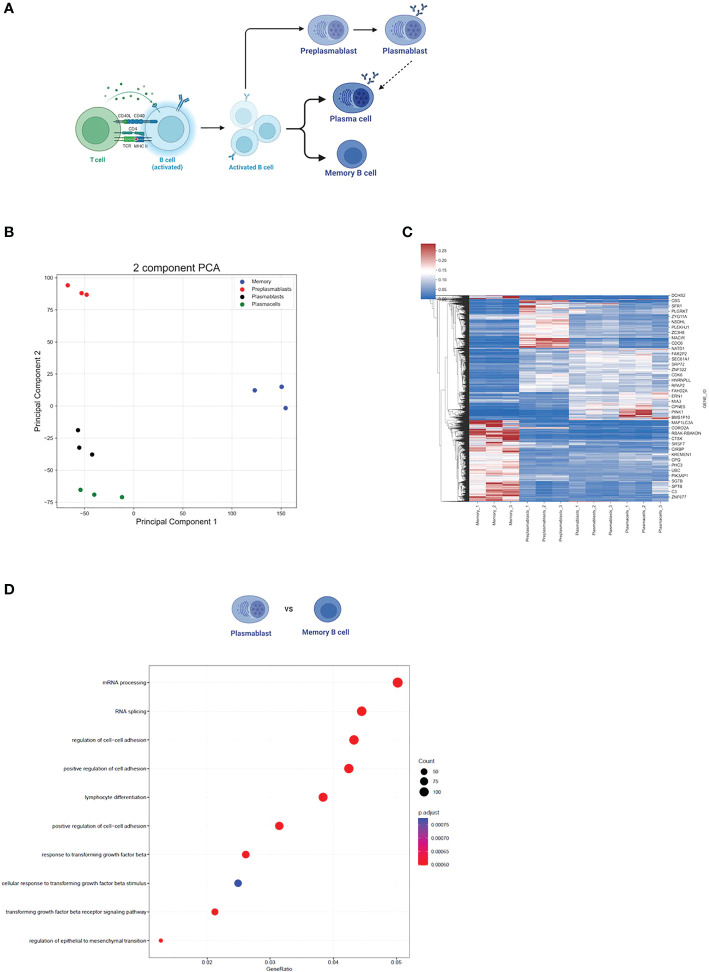
Gene expression during B cell differentiation. **(A)** Summarized schematic representation of the steps of B cell differentiation. Step 1. Antigen recognition induces the expression of effector molecules by T cells, which then activate B cells. Step 2. Activated B-cell proliferation. Step 3. Differentiation to resting memory cells or antibody-secreting cells (PB and PC). Adapted from “Steps in B-cell Differentiation”, by BioRender.com (2022). **(B)** PCA of all genes expressed in at least one subtype of B cells. **(C)** Heatmap showing all differentially expressed genes in B cells. *P*ajd < 0.001, log2FC > 0.5 (or < -0.5). **(D)** The 10 strongest enriched GO terms in PB vs MBC. The GO term enrichment was performed using the enrichGO function (see methods). The size of the dots represents the number of genes in the significant differentially expressed gene list associated with the GO term and the color of the dots represents the *P*-adjusted values (FDR). GeneRatio: the number of differentially expressed genes divided by the total number of genes in the given GO term.

To evaluate differences in gene expression (GE) and alternative splicing (AS) during B cell activation and differentiation, we analyzed publicly available RNA-seq data of preplasmablast (prePB), plasmablast (PB), plasma cells (PC), and memory B cells (MBC) (GSE148924, **Figure 1A**). Our principal component analysis (PCA) of the 15.754 gene expression levels showed that each cell subpopulation was segregated according to its developmental stage and that the triplicate samples of the individual cell types clustered well together (as previously demonstrated in 33) (**Figure 1B**). This analysis rules out high variability or heterogeneity of gene expression within one sample group and we therefore proceed using the triplicates in our analysis. We used DeSeq2 to compare changes in gene expression using a log2FC > 0.5 (< -0.5) and *P*adj *<*0.001 as cut-offs. Overall, we observed a similar gene expression profile between PB and PC (**Figure 1C**), where only 125 genes were differentially expressed between them. We observed a stronger difference when comparing prePB and PB, and prePB and PC (2383 and 3079 differentially expressed genes, respectively). An even stronger difference in gene expression was observed when comparing prePB, PB, and PC to MBC. We identified 6362 differentially expressed genes between prePB and MBC, 6144 differentially expressed genes between PB and MBC, and 4920 differentially expressed genes between PC and MBC (**Figure 1C**).

**Figure 2:**
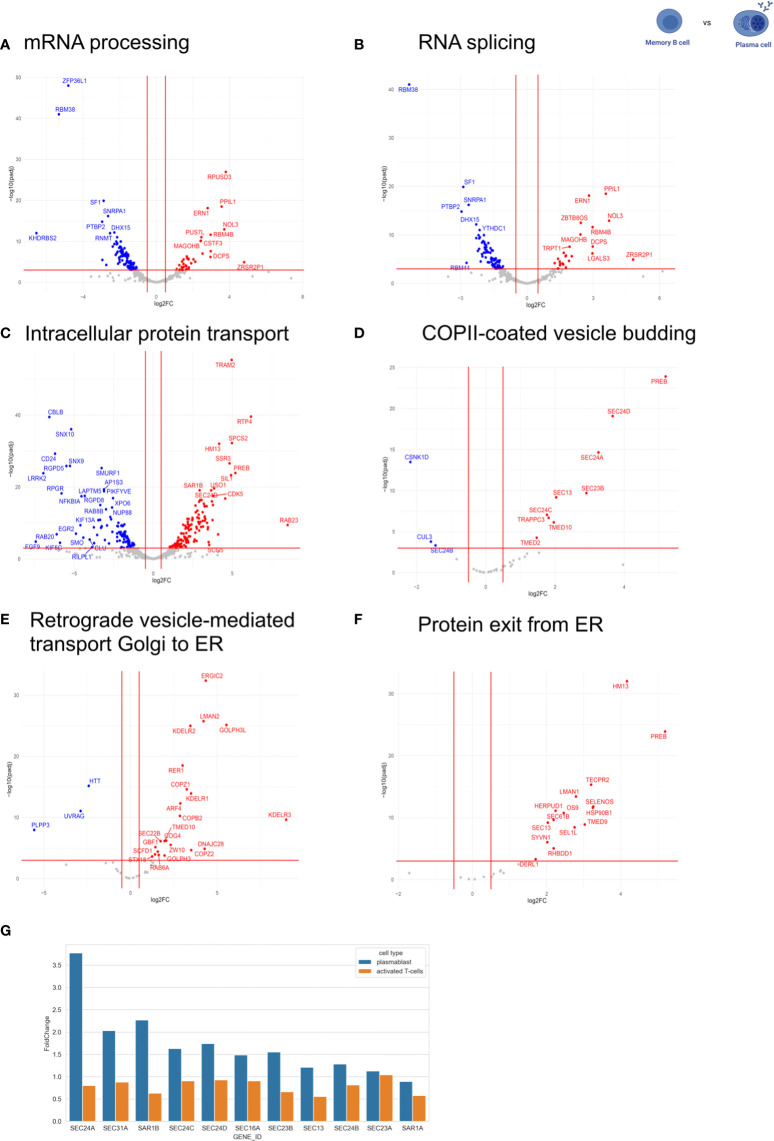
Expression of RNA processing-related and intracellular trafficking-related genes. The expression of RNA processing-related and intracellular trafficking related-genes was compared between MBC and PC. The following GO terms were analyzed: **(A)** mRNA processing. **(B)** RNA splicing. **(C)** intracellular protein transport. **(D)** COPII-coated vesicle budding. **(E)** retrograde vesicle-mediated transport Golgi to ER. **(F)** protein exit from ER. In the volcano plots, log2 fold change (log2FC) was plotted against *P*ajd < 0.001, fold changes were considered significant if log2FC > 0.5 (or < -0.5). Genes up-regulated in plasma cells are shown in red (down-regulated in memory). Genes down-regulated in plasma cells are shown in blue (up-regulated in memory). **(G)** Comparison of gene expression levels of COPII-components in PC compared to prePB and T-cell activation [T-cell data was taken from(34)].

Furthermore, we performed a GO term enrichment of differentially expressed genes amongst the different stages of B cell development. Interestingly, the GO terms mRNA processing (GO:0006397) and RNA splicing (GO:0008380) were highly enriched in MBC when compared to PB (FDR < 0.05) (**Figure 1D**). Although the same GO terms were not enriched when comparing MBC to PC, we observed that for “mRNA processing” 88 out of 410 genes were upregulated in MBC, while only 36 were upregulated in PCs. For “RNA splicing”, 75 genes were upregulated in MBC, and 25 were upregulated in PCs (342 total) (**Figures 2A, B**), suggesting that there is also a substantial difference in mRNA processing and splicing in MBC when compared to PC.

It is well known that PCs are responsible for secreting antibodies, and therefore, have a higher rate of protein biosynthesis than MBCs (4). To analyze whether this is accompanied by an adaptation of the secretory pathway at the level of gene expression, we compared the expression of intracellular trafficking related-genes between MBC and PC by analyzing genes included in the following GO terms: intracellular protein transport (GO:0032779), COPII-coated vesicle budding (GO:0090114), retrograde vesicle-mediated transport Golgi to ER (GO:0048220) and protein exit from ER (GO:0032527) (**Figures 2C–F**). We observed that 431 out of 1257 genes related to intracellular protein transport are differentially regulated between MBC and PC without a significant bias towards up- or down-regulation in either of the two cell types. That is about the expected number, given that the expression of about 1/3 of all expressed genes changes between these B cell subtypes (4920 out of 15.754 genes, see above, **Figure 2C**). However, when we focus on the early secretory pathway by looking at GO terms COPII-coated vesicle budding, Retrograde vesicle-mediated transport Golgi to ER, Protein exit from ER, and ER to Golgi vesicle-mediated transport, we find strong upregulation of many genes almost exclusively in PC (**Figures 2D–F**; **Supplementary Figure 1**). In contrast, the GO-terms trans-Golgi network membrane and Clathrin-related genes are not specifically regulated between the two cell types (**Supplementary Figure 1**). These data indicate that especially the expression of components of the early secretory pathway is upregulated in antibody-secreting B cell subtypes to accommodate the increased secretory cargo flux.

To better understand the mechanism that allows PCs to regulate their secretory machinery, we compared the expression level changes of COPII components in PCs and during the activation of CD4+ T cells (**Figure 2G**) (34). Interestingly, the expression of COPII components during T cell activation stays rather constant, whereas PCs show a substantial upregulation of almost all COPII components when compared to non-antibody-secreting prePB. These data suggest that B cells use changes in gene expression to adjust their secretory machinery during activation and differentiation, whereas T cells rely mostly on alternative splicing of key regulators (34, 35). This may also indicate that B cells require stronger adjustments to their secretory system than T cells, which would make B cell differentiation a very interesting model system to study the adaptation of the secretory pathway in the future.

### Alternative splicing of genes involved in intracellular transport in MBCs

**Figure 3:**
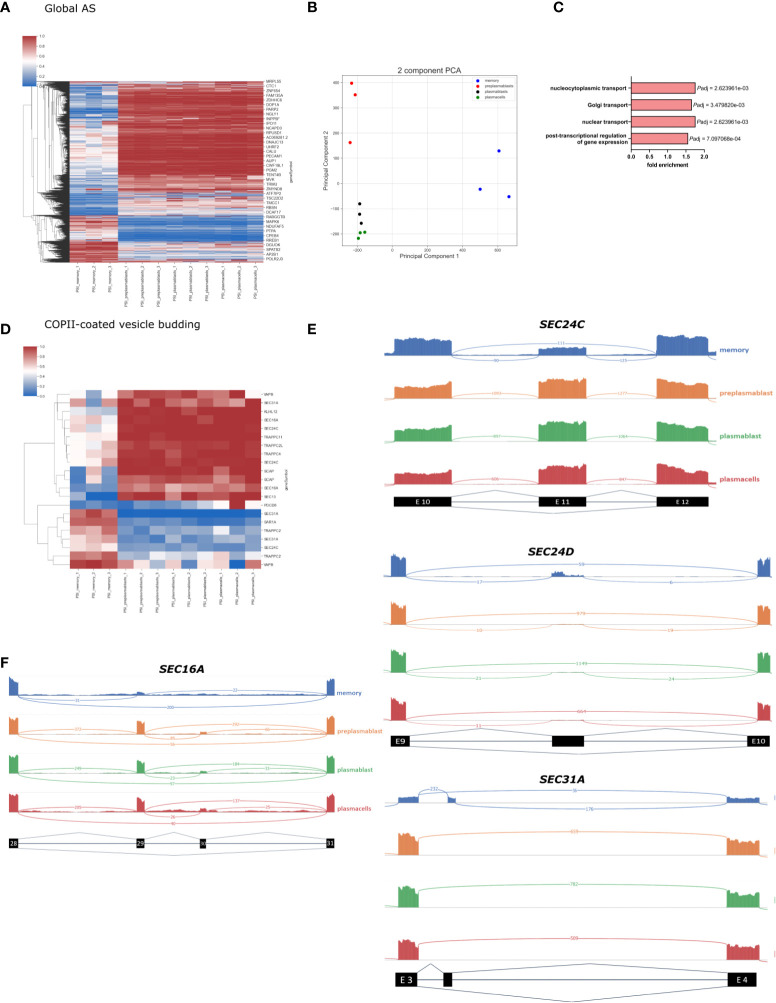
Global splicing analysis in B-Cell differentiation. **(A)** Heatmap showing the PSI of all significantly altered splicing events in B-cell populations (*P-*value < 0.001, DPSI > |0.4|). **(B)** PCA of all annotated splice events in B-cell differentiation. **(C)** Enriched GO terms in AS genes in MBC; only the GO terms related to intracellular transport are shown. *P*-adjusted values (FDR). **(D)** Heatmap showing all significant PSI changes for splicing events in genes in the GO-term “COPII vesicle coat”. **(E)** Sashimi plots for selected frameshift splice events in COPII compartments. **(F)** Sashimi plot showing splicing of SEC16A Exon 29 and 30.

We then performed a global alternative splicing analysis and detected a total of 44.879 AS events, with 3179 events showing significant differences between the differentiation stages we analyzed. All significantly changed alternative splice events are shown in the heatmap in **Figure 3A** (*P*-value < 0.001, DPSI > |0.4|). The significantly changed AS events fall into the following four categories: 2683 (80.07%) exon skipping (SE), 53 (2.16%) intron retention (RI), 209 (8.18%) alternative 5 splice site (A5SS), and 234 (9.58%) alternative 3 splice site (A3SS). As observed in our GE analysis, the AS pattern was rather similar amongst prePB, PB, and PC, while MBC showed substantial differences. The splicing PCA looks similar to the GE PCA, with close relation of PB and PC, which are separated from prePB and MBC (**Figure 3B**). The PCA for splicing events shows a high degree of homogeneity in PB and PC and slightly lower in preBP. Only MBCs show a larger extent of heterogeneity, which is still low considering the distance of the MBC cluster to the other cell types. The vastly different global splicing pattern of MBS is consistent with the gene expression analysis showing enrichment of GO terms related to splicing and RNA processing when comparing genes differentially expressed between MBC and PC (**Figures 2A, B** **)**. Notably, ¾ of all significantly changed splicing events are less included in MBCs, which may point to reduced overall splicing efficiency.

Further, we perform a GO term enrichment analysis of differentially spliced genes in MBC when compared to PCs. In addition to the biological processes involved in post−transcriptional regulation of gene expression, we found an enrichment of the GO terms Golgi vesicle transport (GO:0048193), nuclear transport (GO:0051169), and nucleocytoplasmic transport (GO:0006913), suggesting that genes involved in intracellular transport are heavily alternatively spliced in MBC (**Figure 3C**) (full GO term enrichment analysis in **Supplementary Figure 2**). The global changes in alternative splicing in MBC suggests a substantial contribution in controlling the functional changes during the formation of memory B cells and the specific enrichment of GO terms suggests that alternative splicing plays a fundamental role in controlling intracellular trafficking.

To get a more detailed breakdown of intracellular trafficking, we concentrated on the COPII machinery, which controls the first step in protein secretion. In the GO term COPI-coated vesicle budding, we found 21 alternatively spliced events, which mostly show a MBC unique splicing pattern (**Figure 3D**). Important COPII components like *SEC13*, *SEC31A*, and *SEC24C* have multiple splicing events that are differentially spliced in MBCs compared to the other cell types. For example, *SEC24C* exon 11 is 100% included in prePB, PB, and PC, and only ~50% included in MBC (**Figure 3E**). The length of exon 11 is 116 nucleotides, therefore exclusion of this exon causes a frameshift and a premature stop codon. It is very probable that through nonsense-mediated decay (NMD) this splicing event leads to a lower abundance of SEC24C in MBCs (36), which we detected in our gene expression data in MBCs (**Figure 2D**). SEC24C has a specific IxM cargo binding site and positively regulates the export of the Q-SNARES *Syntaxin5*, *GS27*, and *Bet1* (11). Therefore, the expression level of SEC24C may contribute to cargo specificity in a cell type-specific manner. *SEC24D* also contains an alternatively spliced, frameshift-inducing exon (296 nucleotides in length, after exon 9) that is almost exclusively included in MBC (**Figure 3E**). This again correlates with reduced expression of SEC24D in MBC, suggesting that AS-NMD mediated control of gene expression is a more general mechanism to control components of the trafficking machinery in a cell type-specific manner.

While some COPII components are controlled through NMD-inducing isoforms, other splice variants do not induce a frameshift and NMD and thus can control the functionality of the respective protein. For example, the COPII outer coat component *SEC31A* is heavily alternatively spliced. MBCs include a 50 nucleotides small non-conserved Exon after exon 3. (**Figure 3E**). This alternative exon is likely coupled to an alternative translation start and a shorter N-terminus resulting in the truncation of the first WD40 repeat. The WD40 domain does interact with the WD40 domain of SEC13, the second component of the outer COPII layer (37). Truncation of the *SEC31A* WD40 domain may thus lead to reduced interaction between *SEC31A* and *SEC13* and may control the efficiency of coat formation or coat structure. Structural analysis of COPII coats formed by splice variants of the individual COPII components will be of high interest to address their cell type-specific functionality in future research.

We have previously shown that alternative splicing of the COPII scaffolding protein *SEC16A*, responsible for recruiting the COPII machinery, controls the efficiency of ER export during T cell activation (35). This study demonstrates that *SEC16A* exon 29 is predominantly included in activated T cells, which then increases export efficiency. In B-lymphocytes, we observe a similar splicing pattern. Exon 29 is highly excluded in MBC (PSI = ~15%), whereas in the other differentiation stages it is mostly included (PSI = ~80%) (**Figure 3F**).

**Figure 4:**
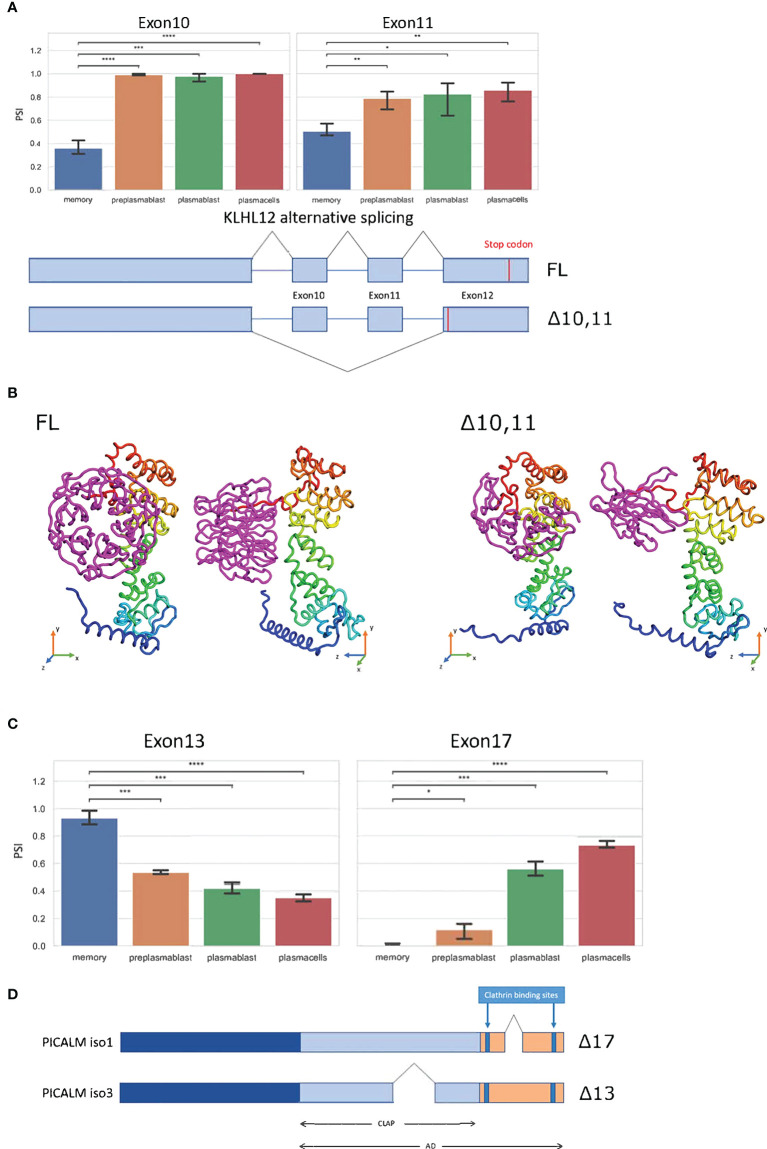
Splice events with protein-coding isoforms. **(A)** Barplot showing PSI of two different splice events in *KLHL12*. Schematic summary of *KLHL12* alternative splicing and the resulting change of stop codon. **(B)** Protein structure prediction with Robetta (https://robetta.bakerlab.org/) of the *KLHL12* isoforms (FL and Δ10,11). The Δ10,11 causes a deletion of the kelch domain (colored magenta) **(C)** Bargraph showing the PSIs of exon 13 and exon 17 of *PICALM* in differentiation stages of B-Lymphocytes. **(D)** Schematic summary of *PICALM* isoforms. Significance is indicated by asterisks (*P < 0.05, **P < 0.01, ***P < 0.001, ****P < 0.0001).

*KLHL12* is likewise alternatively spliced. Here the MBC exclusive isoform is generated through the exclusion of exons 10 and 11 (**Figure 4A**). While exon 10 exclusion with 99 nucleotides would not shift the reading frame, exon 11 has a length of 187 nucleotides, resulting in a frameshift and an alternative stop codon in the last exon, therefore not inducing NMD. Altogether these splice events, if translated, promote a short C-terminus in the *KLHL12* protein (**Figure 4B**). The resulting isoform is missing three out of six kelch repeats, which usually ensure the accurate ubiquitination of SEC31. Without a complete kelch domain, KLHL12 likely is not fully capable of ubiquitinating SEC31, which will decrease the ability to create flexible COPII coats and may result in reduced ability to secrete large cargoes in MBC. Together with the alternative N-terminus of SEC31A discussed above, these splicing-induced changes may lead to substantially altered SEC31A form and function in MBC.

The phosphatidylinositol-binding clathrin assembly protein PICALM regulates clathrin-mediated endocytosis (38) and the size of clathrin-coated vesicles (39). PICALM regulates clathrin assembly through binding clathrin triskelia at its C-terminus, which contains at least two binding sites (40, 41). We find two alternative splice events in *PICALM*, one of them impacts the clathrin binding site. Exon 13 displays higher inclusion in MBCs, and reduced inclusion in prePB, PB, and PC. On the other hand, exon 17 is basically not included in MBC, has a slight inclusion in prePB, and has a PSI of 0.6 and 0.7 in PB and PC, respectively (**Figure 4C**). The Δ17 isoform codes for the Uniprot isoform 1 (Q13492-1) and Δ13 codes for Uniprot isoform 3 (Q13492-3). Isoform 1 has a shorter C-terminal domain, and therefore, is more efficient in bringing two clathrin triskelia together, thus promoting endocytosis. Through alternative splicing, this effect appears to be enhanced in MBC, weaker in prePB and PB and further reduced in PC (**Figure 4D**).

### NERF/ELF2 is likely involved in controlling the expression of genes related to intracellular trafficking in MBCs

**Figure 5:**
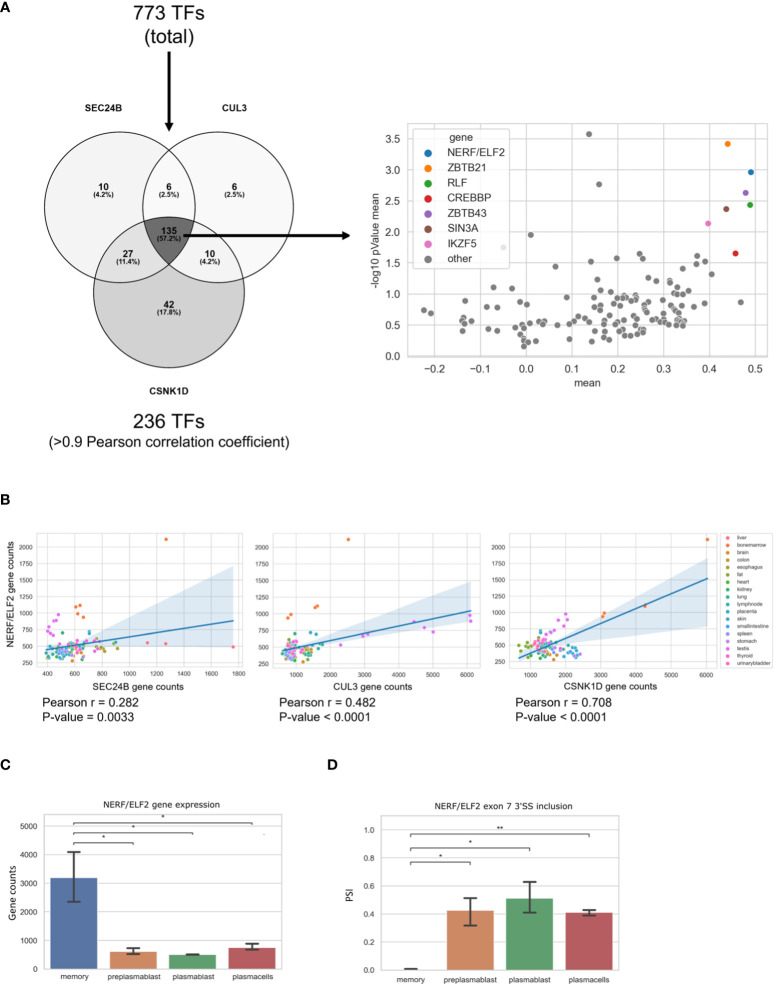
*NERF/ELF2* controls expression of COPII-related genes in MBCs. **(A)** Venn diagram showing the TFs that had a high Pearson correlation coefficient (>0.9) with *SEC24B*, *CUL3*, and *CSNK1D* in MBCs (left). Dotplot showing the Pearson correlation coefficient of the 135 TFs and the *SEC24B*, *CUL3*, and *CSNK1D* genes in publicly available RNA-seq data of 17 human tissues (SRA study ERP003613). Shown in the graph is the mean pearson correlation coefficient against the mean p-value of the correlation in -log10 scale of each TF with the three COPII genes (right). **(B)** Dotplot with added linear regression line for the correlation between *NERF/ELF2* and *SEC24B*, *CUL3*, and *CSNK1D* in human tissue data. Each dot is color coded for originated tissue. **(C)** *NERF/ELF2* gene expression in B cells. **(D)** NERF/ELF2 alternative splicing pattern of Exon 7 3'SS in B cells.

In our analysis, we found three genes (*SEC24B*, *CUL3*, and *CSNK1D*) in the GO-term COPII-coated vesicle budding, which are upregulated in MBCs and are not regulated through alternative splicing. In order to identify a transcription factor that may be responsible for their MBC-specific upregulation, we performed a Pearson correlation analysis. We used a list of transcription factors (TFs) from the GO terms transcription factor binding (GO:0008134) and DNA-binding transcription factor activity, RNA polymerase II-specific (GO:0000981) and calculated the Pearson correlation index between the three COPII genes and all the TFs from the mentioned GO-terms. Using a threshold of the Pearson correlation coefficient of >0.9, we found 236 TFs that are highly correlated with at least one of the three genes. In the next step we identified 135 TFs that highly correlate with all three, *SEC24B*, *CUL3*, and *CSNK1D*, in MBCs. To validate these findings, we performed the same correlation analysis with the 135 TFs and *SEC24B*, *CUL3*, and *CSNK1D* genes in publicly available RNA-seq data from 17 human tissues (SRA study ERP003613). We then averaged the resulting coefficient indices of *SEC24B*, *CUL3*, and *CSNK1D* and each TF. Additionally, the p-values for the correlation of TFs with each of the three genes in all tissues were calculated and averaged (**Figure 5A**). As a result, we found a small group of TFs, whose expression correlates with the expression of *SEC24B*, *CUL3*, and *CSNK1D* in human tissues. The TF with the highest correlation and the lowest *P*-value amongst all tissues was *NERF/ELF2* (**Figure 5B**). We performed a similar analysis for genes in the GO term COPII-coated vesicle budding, which are upregulated in PC but did not find a strong correlation with a single transcription factor (**Supplementary Table 2**). Further, we used the ShinyGO 0.76 tool to detect enriched TF binding motifs in the promoters of genes upregulated in PCs. We detected 30 enriched TF binding motifs, but only two of the corresponding TFs (*TET1* and *E2F6*) were also upregulated in PCs when compared to MBCs (**Supplementary Table 3**). These data suggest that *NERF/ELF2* controls the expression of COPII-related genes in MBC, whereas the specific expression of COPII-related genes in PC is not mediated by altered expression of one individual transcription factor and may be dependent on posttranscriptional or posttranslational mechanisms that can control individual targets or the activity of TFs.

**Figure 6:**
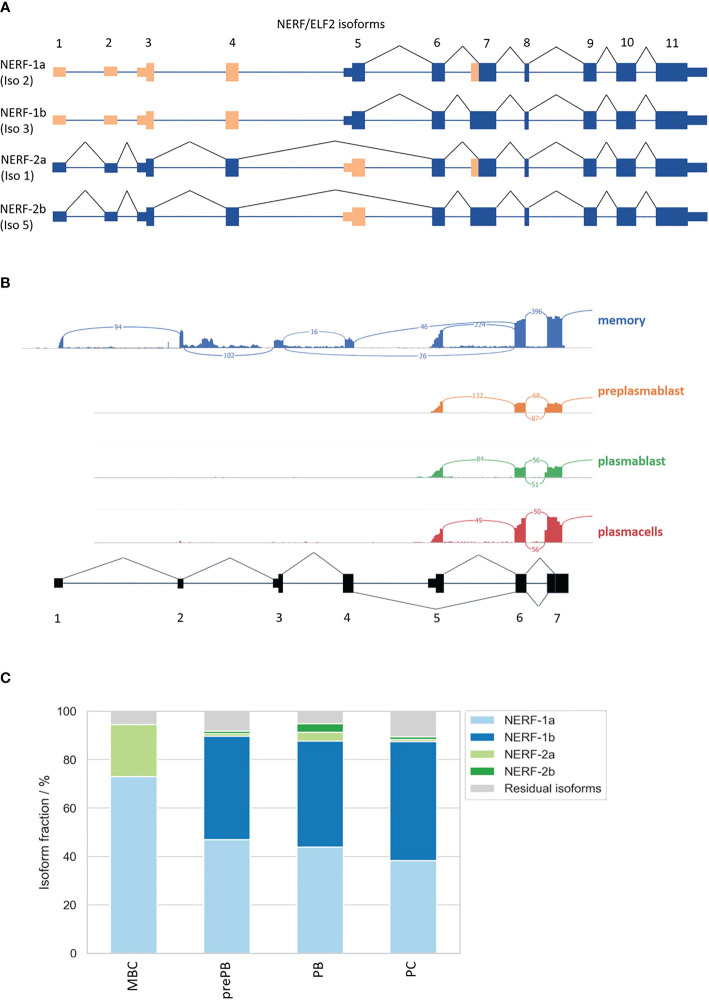
Alternative splicing of the transcription factor *NERF/ELF2*. **(A)** Possible *NERF/ELF2* isoforms according to USCS genome browser and UniProt. **(B)** Sashimi plot of *NERF/ELF2* transcript in MBC, prePB, PB, and PC. **(C)** Stacked barplot of *NERF/ELF2* isoforms in B-Lymphocyte differentiation.

Consistent with the hypothesis that *NERF/ELF2* controls the expression of trafficking-related genes in MBC, the RNA-Seq data show substantially higher expression of *NERF/ELF2* in MBC compared to the other B cell subtypes (**Figure 5C**). In addition to changed gene expression, *NERF/ELF2* is also regulated by alternative splicing, as it is expressed in four main protein-coding isoforms. Both *NERF-1a* and *NERF-1b* isoforms start with exon 5, resulting in a shorter N-terminus. Additionally, *NERF-1a* and *NERF-1b* differ in the use of an alternative 3SS in exon 7 (**Figure 5D**). Similarly, *NERF-2a* and *NERF-2b* differ by the use of the exon 7 alternative 3SS but start with exon 1, which adds a 60 amino acid longer N-terminus (**Figure 6A**). The different splice variants are highly regulated in the different B cell subtypes (**Figure 6B**). In MBCs we observe complete skipping of the exon 7 3SS and some usage of the longer N-terminus leading to the expression of NERF-1a and NERF-2a (**Figure 6C**). In contrast, PrePB, PB, and PC have very similar gene expression levels and splice isoform patterns. Around 90% of their isoforms start with exon 5, therefore mostly expressing the short N-terminus isoforms, which are equally distributed between NERF-1a and NERF-1b (**Figure 6C**). This analysis identifies MBC-specific expression of *NERF/ELF*, regulated at the level of gene expression and alternative splicing, and suggests a role in controlling the MBC-specific expression of intracellular trafficking genes. Future research will address specific functions of different *NERF/ELF* isoforms and a global role in controlling MBC functionality.

## Discussion

We have used publicly available RNA-seq data to address the potential role of AS in regulating intracellular trafficking and secretion during B cell activation and differentiation. We observed clear differences in GE and AS patterns among MBCs and ASCs. Accordingly, there was an enrichment of GO terms related to RNA processing and splicing in MBC when compared to PB. Previous studies have shown that changes in AS events contribute to the regulation of lymphocyte activation (20, 22). However, the role of AS in regulating secretion and intracellular trafficking among the different stages of B cell development still remains to be elucidated.

ASCs are responsible for synthesizing and secreting enormous amounts of Ig molecules (2). To enable an increased capacity of Ig synthesis and secretion, ASCs need to adapt their morphology, metabolism, and gene expression profile (3). In PC, the Golgi expands sixfold in volume, and the ER exit sites increase almost fourfold in number to deal with the increased Ig secretion (5). Consistently, our GE analysis shows that genes related to intracellular trafficking and secretion, including COPII-coated vesicle budding, are upregulated in ASCs when compared to MBCs. More specifically, we provide evidence that the expression of most of the COPII components is upregulated in PC, which likely is required to increase secretory capacity and the number of ER exit sites. Interestingly, PCs express a distinct set of COPII genes, which likely controls the efficiency and specificity of ER export in different B cell subsets. *SEC24A*, *SEC24C*, and *SEC24D* are substantially upregulated in PCs, while only *SEC24B* is upregulated in MBCs. The different variants have binding capabilities to different sets of cargoes and are the active cargo sorter of the COPII machinery (10–12, 42). Altered ratios of SEC24 variants may thus differentially control general export efficiency, e.g. through controlling the export of GPI-anchored proteins (43), but also a preference for specific cargoes.

Furthermore, we observed that MBCs have a different global splicing pattern than the other B cell subtypes. Interestingly, genes related to intracellular transport are significantly differentially spliced in MBC when compared to the other stages of B cell development. Since COPII-coated vesicles are important for packing and exporting Ig molecules (5), we decided to have a closer look at AS events in genes related to COPII. Several AS events in *SEC13*, *SEC24C*, and *SEC24D* were found to be differentially regulated in MBC when compared to ASCs. This suggests that alternative splicing, in addition to changes in gene expression, contributes to adapting the secretory pathway to cell-type-specific requirements. This idea is underlined by our GO term analysis, indicating that several aspects of intracellular transport are controlled at the level of alternative splicing. Another interesting example is *PICALM*, where we observed that the Δ17 isoform is more abundant in MBCs while the Δ13 isoform is more abundant in the other stages of B cell differentiation. *PICALM* is responsible for controlling clathrin-coated vesicle size and maturation, and therefore, determines the rates of endocytic cargo uptake (39). The Δ17 isoform has a shorter C-terminal domain, which facilitates endocytosis and potentially enhances the rate of cargo uptake in MBCs. These analyses together indicate that alternative splicing controls diverse trafficking pathways during B cell activation and differentiation. Furthermore, the adaptation of intracellular trafficking in B cell subsets is different from the situation observed during T cell activation, which largely relies on alternative splicing to control their secretory capacity (34, 35). In addition, AS events that change in COPII genes during T-Lymphocyte activation and B-Lymphocyte differentiation are mostly different, pointing to largely different strategies to adapt the secretory machinery to changing conditions.

Finally, we looked for potential TFs that could be responsible for regulating the expression of *SEC24B*, *CUL3*, and *CSNK1D* in MBCs. We found a strong correlation between the expression of the TF NERF/ELF2 and the expression of *SEC24B*, *CUL3*, and *CSNK1D* in MBCs. Additionally, we found several isoforms of NERF/ELF2 differently expressed during the stages of B cell differentiation. It has been previously reported that the *NERF-2* isoform is responsible for activating the expression of its target genes, while *NERF-1* functions as a transcription repressor (44). Additionally, *NERF-1* isoform overexpression reduced cell proliferation and induced apoptosis, whereas *NERF-2* isoforms did not have these negative regulatory effects (45). The same study demonstrated that the ratio of *NERF-1* to *NERF-2* changes in CD4 and CD8 T cells and early B-cell maturation. From our analysis, it becomes apparent that in mature B-Lymphocytes only MBCs express *NERF-2* isoforms in a substantial manner. PrePB, PC, and PC express more of the apoptosis-inducing isoform *NERF-1* while *NERF-2* is only marginally expressed. The increased *NERF-2* expression in MBCs may contribute to the long lifespan of these cells, which may not be required for other, shorter-lived B cell subtypes.

In summary, we provide evidence for a cell type-specific regulation of intracellular trafficking pathways during lymphocyte activation and differentiation that is mediated at the level of gene expression and alternative splicing. We identify NERF/ELF2 as a potential transcription factor involved in controlling the expression of genes involved in the early secretory pathway and provide evidence for several cell type-specific splice variants of the COPII machinery. These hypotheses are the result of an *in silico* approach and clearly require wet lab follow up, to experimentally validate the impact of alternative splicing, e.g. of COPII components, in regulating cell type-specific intracellular trafficking during lymphocyte activation and differentiation.

## Data availability statement

The datasets presented in this study can be found in online repositories. The names of the repository/repositories and accession number(s) can be found below: www.ncbi.nlm.nih.gov/geo, GSE148924. www.ncbi.nlm.nih.gov/bioproject/, PRJEB4337.

## Author contributions

FO performed RNA-seq analysis with the help of BL. FO, BL, and FH analyzed and interpreted data and wrote the manuscript. FH initiated and supervised the project. All authors contributed to the article and approved the submitted version.

## Funding

Funding was provided by the Collaborative Research Centre 958 “Scaffolding of Membranes - Molecular Mechanisms and Cellular Functions” (SFB 958), project A21.

## Acknowledgments

The authors thank the HPC Service of ZEDAT, Freie Universität Berlin, for computing time. We thank members of the Heyd laboratory for critical discussions. We also thank Dr. Marco Preußner and Dr. Alexander Neumann for their help with RNA-seq analysis. BL thanks the International Max Planck Research School for Biology AND Computation (IMPRS-BAC) for funding.

## Conflict of interest

The authors declare that the research was conducted in the absence of any commercial or financial relationships that could be construed as a potential conflict of interest.

## Publisher’s note

All claims expressed in this article are solely those of the authors and do not necessarily represent those of their affiliated organizations, or those of the publisher, the editors and the reviewers. Any product that may be evaluated in this article, or claim that may be made by its manufacturer, is not guaranteed or endorsed by the publisher.

## Supplementary material

Supplementary Table 1 RNA-seq analysis using DEseq and rMATS. DEseq gene counts ar displayed for each sample and each detected gene. RMATS quantification shows the significant splicing events. Gene name, the coordinates of the splice event, the type of splice event (SE: skipped exon, RI: intron retention, A5SS: alternative 5 splice site, A3SS: alternative 3 splice site) and the PSI for every sample.

Supplementary Table 2 Correlation analysis of PC upregulated COPII vesicle coat budding genes in human tissues. For every gene the pearson R and the p-value of correlation for all nine genes upregulated in PC in the GO term COPII-coated vesicle budding are displayed.

Supplementary Table 3 Transcription factor binding motifs enriched in the promoter of all genes upregulated in PCs. The analysis was performed with ShinyGO 0.76 tool using a list of 2746 genes upregulated in PCs when compared to MBCs. ShinyGO retrieves enriched TF binding motifs, transcript annotation, and promoter sequences from Ensembl. This tool recorded the best score for each of the TFs in every 300bp and 600bp promoter sequence. Students t-test is used to compare the scores observed in a group of genes against the rest of the genes. The *P*-values are corrected for multiple testing using FDR.
