# Supplementary material for: In silico analysis of alternative splicing events implicated in intracellular trafficking during B-lymphocyte differentiation
Source: Front Immunol. 2022 Nov 10;13:1030409. doi: 10.3389/fimmu.2022.1030409 (PMC9691287; doi:10.3389/fimmu.2022.1030409)
Supplement: Supplementary Table 1 — RNA-seq analysis using DEseq and rMATS. DEseq gene counts ar displayed for each sample and each detected gene. RMATS quantification shows the significant splicing events. Gene name, the coordinates of the splice event, the type of splice event (SE: skipped exon, RI: intron retention, A5SS: alternative 5 splice site, A3SS: alternative 3 splice site) and the PSI for every sample. [file DataSheet_1.pdf]

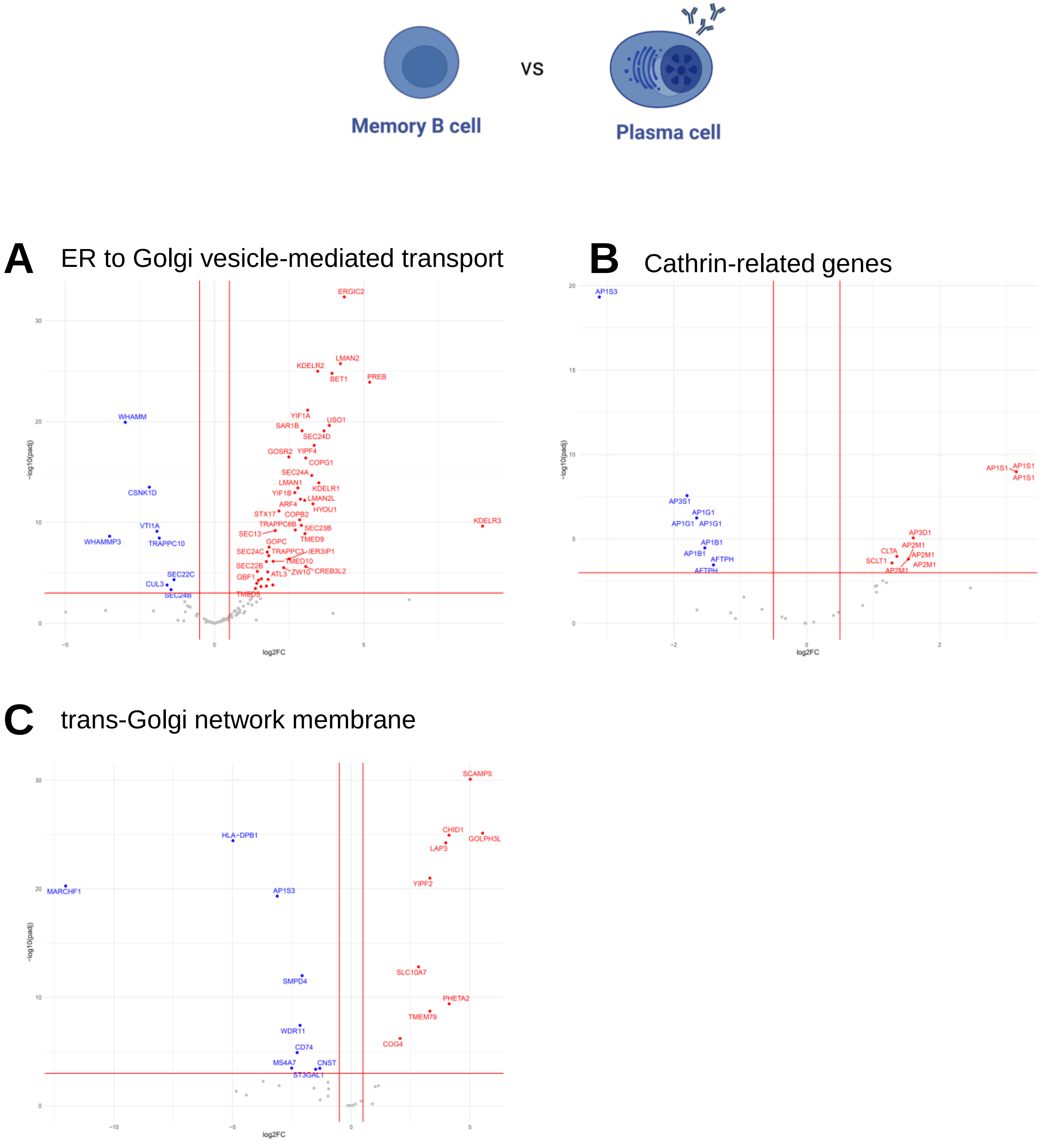

Supplementary Figure 1. Expression of intracellular transport-related genes.

The expression of intracellular transport related genes was compared between MBC and PC. The following GO terms were analyzed: **(A)** ER to Golgi vesicle-mediated transport (GO:0006888) **(B)** Clathrin-related genes (taken from the GO terms: “clathrin adaptor complex - GO:0030131”, “clathrin-coated vesicle cargo loading - GO:0035652”, and “clathrin complex - GO:0071439”). **(C)** trans-Golgi network membrane (GO:0032588). In the volcano plots, log2 fold change was plotted against *P*ajd < 0.001, fold changes were considered significant if log2FC > 0.5 (or < -0.5). Genes up-regulated in plasma cells are shown in red (down-regulated in memory). Genes down-regulated in plasma cells are shown in blue (up-regulated in memory).

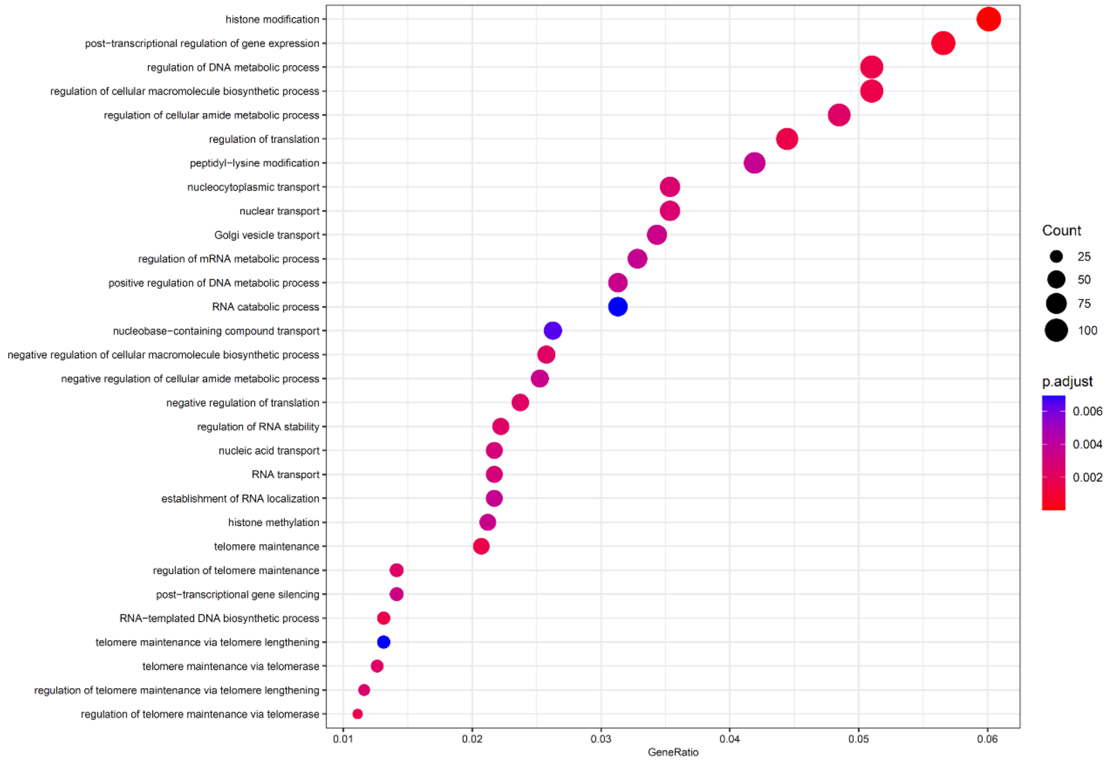

Supplementary Figure 2. GO term enrichment analysis of genes wit significantly changed alternative splice events between MBC and PC

The 30 strongest enriched GO terms in PC vs MBC. The GO term enrichment was performed using the enrichGO function (see methods). The size of the dots represents the number of genes in the significant differentially expressed AS list associated with the GO term and the color of the dots represents the *P*-adjusted values (FDR). GeneRatio: the number of differentially expressed genes divided by the total number of genes in the given GO term.

Supplementary Table 1. Data generated by the RNA seq analysis with DEseq and rMATS.

Supplementary Table 2. Correlation analysis of MBC upregulated "COPII vesicle coat" genes in human tissues.
